# Visualization of Procollagen IV Reveals ER-to-Golgi Transport by ERGIC-independent Carriers

**DOI:** 10.1247/csf.20025

**Published:** 2020-06-18

**Authors:** Yuto Matsui, Yukihiro Hirata, Ikuo Wada, Nobuko Hosokawa

**Affiliations:** 1 Laboratory of Molecular and Cellular Biology, Institute for Frontier Life and Medical Sciences, Kyoto University, Kyoto 606-8507, Japan; 2 Department of Cell Sciences, Institute of Biomedical Sciences, Fukushima Medical University School of Medicine, Fukushima 960-1295, Japan

**Keywords:** collagen, procollagen IV, endoplasmic reticulum, ER-to-Golgi transport, ERGIC

## Abstract

Collagen is the most abundant protein in animal tissues and is critical for their proper organization. Nascent procollagens in the endoplasmic reticulum (ER) are considered too large to be loaded into coat protein complex II (COPII) vesicles, which have a diameter of 60–80 nm, for exit from the ER and transport to the Golgi complex. To study the transport mechanism of procollagen IV, which generates basement membranes, we introduced a cysteine-free GFP tag at the N-terminus of the triple helical region of the α1(IV) chain (cfSGFP2-col4a1), and examined the dynamics of this protein in HT-1080 cells, which produce endogenous collagen IV. cfSGFP2-col4a1 was transported from the ER to the Golgi by vesicles, which were a similar size as small cargo carriers. However, mCherry-ERGIC53 was recruited to α_1_-antitrypsin-containing vesicles, but not to cfSGFP2-col4a1-containing vesicles. Knockdown analysis revealed that Sar1 and SLY1/SCFD1 were required for transport of cfSGFP2-col4a1. TANGO1, CUL3, and KLHL12 were not necessary for the ER-to-Golgi trafficking of procollagen IV. Our data suggest that procollagen IV is exported from the ER via an enlarged COPII coat carrier and is transported to the Golgi by unique transport vesicles without recruitment of ER-Golgi intermediate compartment membranes.

## Introduction

Collagen is the most abundant protein in animal tissues, and 28 types of collagen have been identified in human (Ricard-Blum, 2011). Collagens create frameworks in the extracellular region to organize tissues, such as bones, skin, and organs, as well as networks for cells and other molecules to direct cell migration. Newly synthesized procollagen α-chains are translocated into the ER as a monomer and then a triple helix forms in three α-chains from the C-terminus in a zipper-like manner (Davis *et al.*, 1989; Dölz *et al.*, 1988). Hydroxylation of proline residues stabilizes the triple helix by forming hydrogen bonds and this is important for secretion of procollagens (Berg and Prockop, 1973; Jimenez *et al.*, 1973; Rosenbloom *et al.*, 1973). Ascorbic acid is required as a co-factor of prolyl-4-hydroxylase during this process (Mussini *et al.*, 1967). Collagen IV is a network-forming collagen and a major component of basement membranes. A trimer of α1(IV)_2_α2(IV) is present in all basement membranes, and the length of procollagen IV is ~400 nm (Bachinger *et al.*, 1982).

In the conventional secretory pathway, coat protein complex II (COPII)-coated vesicles carry newly synthesized secretory and membrane proteins from the ER to the Golgi complex (Barlowe *et al.*, 1994; Jensen and Schekman, 2011). COPII vesicles budding from ER exit sites (ERES) assemble into the ER-Golgi intermediate compartment (ERGIC), which moves along microtubules toward the Golgi complex or serves as a stationary sorting station (Appenzeller-Herzog and Hauri, 2006; Bannykh *et al.*, 1996; Ben-Tekaya *et al.*, 2005; Presley *et al.*, 1997; Scales *et al.*, 1997; Stephens *et al.*, 2000). COPII coatomer Sec13 (Schmidt *et al.*, 2013; Townley *et al.*, 2008; Townley *et al.*, 2012), Sec24D (Ohisa *et al.*, 2010; Sarmah *et al.*, 2010), and Sec23A (Boyadjiev *et al.*, 2006; Lang *et al.*, 2006; Zhu *et al.*, 2015) are essential for collagen export, and a defect in Sar1 impairs transport of procollagens I and VII (Cutrona *et al.*, 2013; Mironov *et al.*, 2003; Nogueira *et al.*, 2014; Stephens and Pepperkok, 2002). However, typical COPII vesicles are 60–90 nm in diameter (Bannykh *et al.*, 1996; Barlowe *et al.*, 1994) and thus are too small to accommodate procollagen trimers of ~300–400 nm in length, including procollagen IV (Bachinger *et al.*, 1982; Malhotra and Erlmann, 2015; Malhotra *et al.*, 2015).

Two basic modes of ER-to-Golgi transfer have been proposed to explain the export of large and rigid procollagen molecules; one is mediated by large carriers that can accommodate the procollagen trimers and the other bypasses vesicular transport. In addition, ER-to-Golgi transport of procollagen I is reportedly achieved by a transport complex in a similar manner to trafficking of conventional secretory cargo (Stephens and Pepperkok, 2002). Alternatively, unique tubules or long tubular vesicles may be involved in the transport of procollagen I (Bonfanti *et al.*, 1998; Mironov *et al.*, 2003). TANGO1, a transmembrane protein located at ERES, helps to enlarge COPII vesicles by interacting with collagen VII and COPII coatomer (Saito *et al.*, 2009). Subsequent studies revealed that megacarriers, the diameter of which sometimes exceeds 1 μm, form upon recruitment of ERGIC membranes to ERES by TANGO1 for transport of collagen VII (Malhotra and Erlmann, 2015; Malhotra *et al.*, 2015; Nogueira *et al.*, 2014; Santos *et al.*, 2015). In *Drosophila*, Tango1 increases secretory capacity by maintaining Golgi proximity and rebuilding larger ERES via multiple interactions of this protein with other molecules (Liu *et al.*, 2017). On the other hand, a recent study reported direct ER-to-Golgi transfer of procollagens without vesicular transport. This “short-loop transport” pathway was identified by live-cell imaging of procollagen I (McCaughey *et al.*, 2019).

Recent studies revealed that additional unique factors are required for ER-to-Golgi transport of procollagens in addition to TANGO1. The CUL3-KLHL12 complex monoubiquitinates Sec31 coatomer to enlarge COPII vesicles such that they can accommodate procollagen I (Gorur *et al.*, 2017; Jin *et al.*, 2012). Sedlin, a TRAPPII complex component, promotes the GTPase cycle of Sar1, which is required for export of procollagens from the ER (Cutrona *et al.*, 2013; Mironov *et al.*, 2003; Nogueira *et al.*, 2014; Stephens and Pepperkok, 2002), for transport of procollagen I and II (Venditti *et al.*, 2012). This observation suggests that large COPII vesicles can form via modulation of the GTPase cycle (Saito and Katada, 2015; Saito *et al.*, 2017). SCFD1/SLY1, which regulates membrane fusion and intracellular transport (Dascher and Balch, 1996), is involved in export of procollagen VII, but not of procollagen I, through interactions with TANGO1 and the ER t-SNARE Syntaxin 18 (Nogueira *et al.*, 2014). TFG is another class of protein that is thought to form meshwork tethers for Sec23 and thereby facilitate juxtaposition of COPII-coated carriers and the ERGIC (Johnson *et al.*, 2015). A recent study revealed that TFG supports the budding of COPII vesicles containing procollagen I (McCaughey *et al.*, 2016). NRZ (NBAS, RINT1, ZW10) proteins are also involved in collagen VII transport by tethering ERGIC53 to TANGO1 at ERES (Raote *et al.*, 2018).

In this study, to elucidate the molecular mechanisms underlying export of procollagen IV, we tracked the intracellular trafficking of this protein by performing live-cell imaging. We visualized ER-to-Golgi transport of procollagen IV using cysteine-free SGFP2-tagged proα1(IV) chain (cfSGFP2-col4a1). Live-cell imaging revealed that collagen IV was transported in vesicles with a diameter of ~300–500 nm, which were a similar size as carriers containing GFP-labeled α_1_-antitrypsin (α1AT). However, cfSGFP2-col4a1-containing vesicles did not co-localize with ERGIC53 or RAB1 until they fused with the Golgi complex, suggesting that they did not recruit ERGIC membranes. Knockdown of Sar1 and SLY1/SCFD1 inhibited ER-to-Golgi transport of procollagen IV; however, knockdown of TANGO1, CUL3, and KLHL12 did not. These results suggest a unique mechanism for the exit of procollagen IV from the ER.

## Materials and Methods

### Plasmid construction

cfSGFP2-col4a1 was constructed by inserting cysteine-free SGFP2 (Suzuki *et al.*, 2012) with a flexible linker at the N-terminus of the triple helical region, which was downstream of the propeptide (7S domain), of the α1 chain of procollagen IV. Complementary DNA was isolated by PCR amplification from a cDNA library prepared from HeLa cells. Restriction sites (NheI at the 5' flanking sequence and SacI at the 3' flanking sequence) were attached to the COL4A1 fragment encoding the N-terminal region (Met1–Pro184), including the signal sequence, 7S domain, and the first 12 amino acids of the triple helical region, and ligated into the corresponding sites of the cfSGFP2-N vector in which a flexible linker sequence encoding RSTGSTGSTGA had been inserted between the EcoRI and KpnI sites. Thereafter, the rest of the mature C-terminal domain of COL4A1 (Pro187–Thr1669) was inserted between the BsrGI and HpaI sites of this vector.

To generate PDI-mCherry, the KDEL ER-retrieval sequence was introduced at the C-terminus of mCherry, and then PDI lacking ER-retrieval signals were inserted at the N-terminus of mCherry. A DNA fragment encoding CFP from pECFP-Golgi (Clontech) was replaced by BFP to create Golgi-BFP. To construct mCherry-ERGIC53, mCherry lacking both the N-terminal initiation codon and C-terminal stop codon as well as ERGIC53 lacking a signal sequence were PCR-amplified and sequentially subcloned into the pCMV-Tag4C vector (Stratagene, La Jolla, CA) containing the signal sequence of α1AT. To generate GFP-α1AT, α1AT was subcloned into the pCMV-Tag4C vector, and EGFP was inserted after the signal sequence cleavage site of α1AT at the endogenous BamHI site. mScarlet-RAB1A was constructed by subcloning RAB1A cDNA into the mScarlet-N1 vector. Construction of mScarlet-RAB1B was described elsewhere (Hirata *et al.*, manuscript in preparation).

To construct the shRNA vector p7SKU6iRton, which was used to knockdown two target mRNAs simultaneously and label cells expressing the plasmid with the near infrared fluorescent protein iRFP670 (Shcherbakova and Verkhusha, 2013), we combined three expression cassettes driven by the human U6, human 7SK, and thymidine kinase promoters. The former two polymerase III promoters were used to drive the shRNA, and the latter was to express the fluorescent tag. The 23-mer double-stranded shRNA oligonucleotides with six consecutive thymidine residues were inserted into the BstZ17I site downstream of the TATA box of the human U6 promoter or into the EcoRV site of the human 7SK promoter, which was placed at the consensus transcription initiation position such that the 5' proximal G nucleotide of the inserted double-stranded oligonucleotide became the actual initiation site and only the designed double-stranded RNA was released from Dicer. DNA fragments encoding the human 7SK and human U6 promoters as well as the herpesvirus thymidine kinase promoter were synthesized (Integrated DNA Technologies, Coralville, IO). The coding region of iRFP670 was PCR-amplified from piRFP670-N1 (Addgene, Cambridge, MA) and that of SBFP2 (Kremers *et al.*, 2007) was synthesized (Integrated DNA Technologies). Each functional unit was combined by Gibson Assembly or NEBuilder (NEB Japan, Tokyo, Japan) and embedded into pSilencer 1.0-U6 (Ambion, Austin, TX). The sequences of the double-stranded oligonucleotides inserted at the BstZ17I and EcoRV sites were as follows: Sar1a, 5'-GTTCTCGCCTCGTGGAATCCAAAGCGAACTTTGGATTCCACGAGGCGAGAATTTTTT-3'; and Sar1b, 5'-GCTAATGTGCCTATACTGATTCGAAAATCAGTATAGGCACATTAGCTTTTTT-3'. For the construction of shRNA targeting KLHL12 #1, p7SKU6iRn, in which the restriction enzyme site BstZ17I of p7SKU6iRton was replaced by BbsI, was used. The oligonucleotide sequence inserted was: 5'-GAATCCTTGCCTAACCTGCTACATCAAGAGTGTAGCAGGTTAGGCAAGGATTC-3'. For shRNAs targeting TFG and KLHL12 #2, the sequences of the oligonucleotides inserted at the BstZ17I of p7SKU6iRton were: TFG, 5'-GGCTCCTCTACACCAATTAATCGAAATTAATTGGTGTAGAGGAGCCTTTTTT-3'; and KLHL12 #2, 5'-GCCTAGATGATTGGAAAGATACGAATATCTTTCCAATCATCTAGGCTTTTTT-3'. Oligonucleotide sequences for shRNAs targeting CUL3, SLY1/SCFD1, and RINT1 were inserted into p7SKU6SBpur, a derivative of p7SKU6iRton, in which the open reading frame of iRFP670 was replaced with that of SBFP2. The sequences of oligonucleotides were: CUL3, 5'-GGTGATGATTAGAGACATACTAACGAATTAGTATGTCTCTAATCATCACCTTTTTT-3'; SLY1/SCFD1 5'-GAGACTTATTGATCTCCATACAAACGAA TTTGTATGGAGATCAATAAGTCTTTTTT-3'; and RINT1, 5'-GGTTATAACTGACAGGTATCGAAATACCTGTCAGTTATAACCTTTTTT-3'.

For the construction of shRNA-resistant SAR1A, SAR1B, and SLY1/SCFD1, codons in the shRNA sequences were replaced as follows: SAR1A, 5'-CAGCAGATTGGTCGAGAGTAAGG-3'; SAR1B, 5'-CCAACGTCCCCATCTTAATC-3'; and SLY1/SCFD1, 5'-CGGTTGATCGACTTGCACACCAA-3'. Synthesized DNA fragments of SAR1A and SAR1B (gBlocks, Integrated DNA Technologies) were subcloned into pcDNA3.1+ plasmid using NEBuilder. To generate shRNA-resistant SLY1/SCFD1, mutations were introduced by inverse PCR using KOD One DNA polymerase (TOYOBO, Japan).

### siRNA oligos

The siRNA oligo sequences used were: TANGO1, 5'-GAUAAGGUCUUCCGUGCUUTT-3' (Santos *et al.*, 2015); BET3, 5'-UCACUCCAAGCAUUACUAAUUTT-3' (Yu *et al.*, 2006); CUL3, 5'-GGUGAUGAUUAGAGACAUACUAATT-3'; KLHL12, 5'-GAAUCCUUGCCUAACCUGCUACATT-3'; and TFG, 5'-ACUUCUGAGUAAUGAUGAATT-3' (McCaughey *et al.*, 2016). Medium GC negative control siRNA (Invitrogen) was used as a control siRNA.

### Antibodies

A rabbit polyclonal antibody against the NC1 domain of the human collagen IV α1 chain was kindly provided by Dr. Sasaki (Oita University, Japan). This antibody recognized human collagen IV on Western blots (Suppl. Fig. 1) and immunoprecipitation (Suppl. Fig. 2A). The following commercially available antibodies were also used: mouse anti-GFP (Roche), mouse anti-MIA3/TANGO1 (Santa-Cruz Biotechnology), mouse anti-CUL3 (Santa-Cruz Biotechnology), mouse anti-KLHL12 (Santa-Cruz Biotechnology), mouse anti-TFG (Proteintech), rabbit anti-SLY1/SCFD1 (CUSABIO Technology), rabbit anti-RINT1 (Proteintech), rabbit anti-TRAPPC3/BET3 (CUSABIO Technology), rabbit anti-Sar1A (Proteintech), rabbit anti-Sar1B (Proteintech), mouse anti-actin (Millipore), rabbit anti-calnexin (Enzo Life Sciences), horseradish peroxidase-conjugated rabbit anti-mouse IgG (Invitrogen, Waltham, MA), horseradish peroxidase-conjugated goat anti-rabbit IgG (Biomedical Technologies, Madrid, Spain), Alexa Fluor 594-conjugated goat anti-rabbit IgG (Thermo Fisher, MS), and Alexa Fluor 488-conjugated goat anti-mouse IgG (Thermo Fisher).

### Cell culture, transfection, and drug treatment

The HT-1080 human sarcoma cell line (ATCC; CCL-121, provided by Dr. Klaus Kühn, Max-Plank Institute, Germany) was grown in Dulbecco’s Modified Eagle’s Medium (Sigma-Aldrich, St Louis, MO) supplemented with 10% fetal bovine serum (Gibco, Waltham, MA), penicillin (10 U/μl), and streptomycin (10 μg/μl), and kept free from mycoplasma and other microorganisms. Cells were transfected using TransIT (TAKARA, Otsu, Japan) according to the manufacturer’s instructions. cfSGFP2-col4a1 and shRNA-expressing plasmids were transfected ~48 h prior to live-cell imaging unless stated otherwise in the legends. For the siRNA transfection, Lipofectamine RNAi-MAX (Invitrogen) was used according to the manufacturer’s instructions. To induce ER-to-Golgi trafficking of cfSGFP2-col4a1, ascorbic acid phosphate (WAKO, Osaka, Japan) was added to the medium at a final concentration of 136 μg/ml.

### Live-cell imaging

Cells were grown on 35 mm glass-bottom dishes (MATSUNAMI, Osaka, Japan) and transfected with plasmids. Images were acquired using a Leica TCS SP8 confocal microscope (Leica Microsystems, Wetzler, Germany) equipped with a 63×/1.4 N.A. oil immersion objective or with a DMI6000B fluorescence microscope (Leica) equipped with a 40×/0.75 N.A. objective. Images were analyzed with LAS AF (Leica Microsystems). Filter sets and confocal settings were optimized for each fluorescent protein. The temperature was maintained at 37°C with 5% CO_2_ using a microscope incubator (TOKAI HIT CO., LTD, Shizuoka, Japan).

Z stacks were acquired using optimized settings and reconstructed using LAS AF 3D viewer. To measure the sizes of cfSGFP2-col4a1- and GFP-α1AT-containing vesicles (Fig. 2A), images (1024×1024 pixels) were acquired using a Leica TCS SP8 microscope including the LIGHTNING package with a 60×/1.4 NA oil immersion objective. This setup has ~140 nm resolution when the pinhole is narrowed at 0.5 Airy Unit and the images are processed by deconvolution. Vesicles were identified and their diameters were measured manually. For photo-bleaching, a 100% laser beam was focused on the intended area. Images were acquired immediately before and after photo-bleaching using a photomultiplier tube. Time-lapse images were acquired after changing the setting to HyD or lightning mode. Line graph software in LAS AF (Leica) was used to identify vesicles containing two different fluorescent proteins.

### Immunocytochemistry

Cells were prepared and stained with anti-collagen IV (1/500) and anti-GFP (1/500) antibodies as described previously (Hosokawa and Wada, 2016). Images were acquired using a Leica TCS SP8 confocal microscope.

### Western blotting

Culture medium was collected, centrifuged at 500×g for 5 min, and mixed with Laemmli’s buffer. Cells were lysed in buffer containing 1% NP-40, 50 mM Tris-HCl (pH 7.6), 150 mM NaCl, and 5 mM EDTA, and supplemented with protease inhibitors (0.2 mM AEBSF, 2 mM NEM, 1 μg/ml leupeptin, and 1 μg/ml pepstatin). Cell lysates were centrifuged at 13,000×g for 20 min at 4°C and the supernatant was mixed with Laemmli’s buffer. After separation by 6% SDS-PAGE, samples were transferred to a nitrocellulose membrane (GE Healthcare, IL) or PVDF membrane (Millipore) and blotted with specific antibodies. Signals were detected using Pierce Western blotting substrate (Thermo Scientific, MS) and visualized with the LAS-4000 Chemiluminescence & Fluorescence Imaging System (GE Healthcare) as described previously (Hosokawa and Wada, 2016). Signals were quantified using ImageQuant (GE Healthcare).

### Statistical analyses

Statistical significance was determined by the Student’s t-test. A difference in means was considered statistically significant at P<0.05 or P<0.01, as indicated in the figures. Error bars depict the SEM.

## Results

### cfSGFP2-col4a1 is transported from the ER to the extracellular region via the Golgi complex

**Fig. 1:**
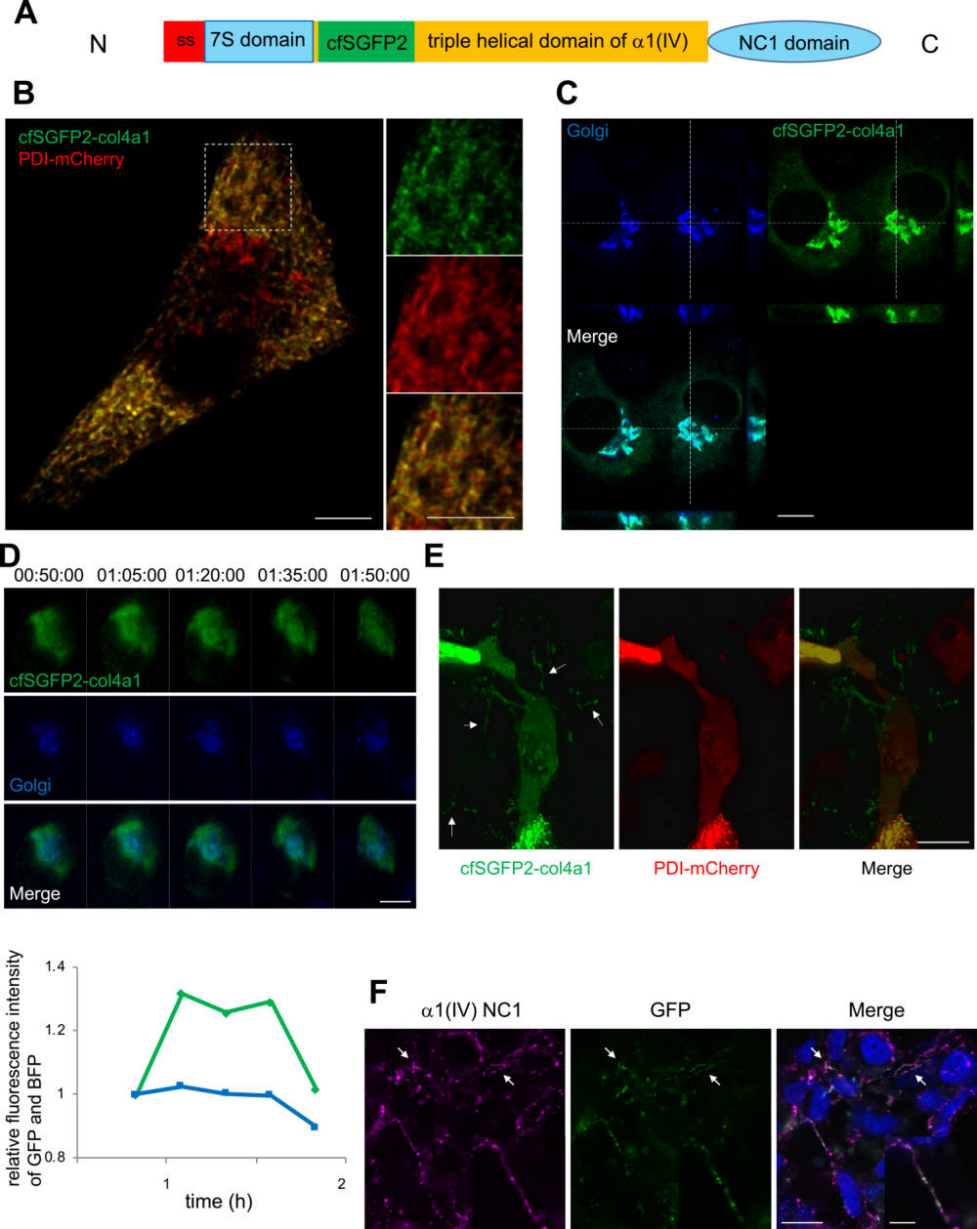
Live-cell imaging of cfSGFP2-col4a1 transport from the ER to the extracellular region. (A) Schematic representation of cfSGFP2-col4a1. cfSGFP2 was inserted at the N-terminus of the triple helical region of the proα1(IV) chain. ss, signal sequence; N, N-terminal; C, C-terminal. (B) Live-cell imaging of HT-1080 cells transiently expressing cfSGFP2-col4a1 (green) and PDI-mCherry (red) by confocal microscopy at 24 h after addition of ascorbic acid. Panels on the right show higher magnification images of the white dotted boxed region. Scale bar, 5 μm. (C) Same as in (B), except cells co-expressing cfSGFP2-col4a1 (green) and Golgi-BFP (blue) were imaged at 2 h after addition of ascorbic acid. Z-stacks at the vertical dotted line (y-z) and horizontal line (x-z) are shown on the right and lower sides, respectively. Scale bar, 10 μm. (D) Time-lapse images of cells co-expressing cfSGFP2-col4a1 (green) and Golgi-BFP (blue) acquired by fluorescence microscopy. The graph shows the relative fluorescence intensities of GFP (green) and BFP (blue) at the Golgi region circled by white doted box. Data are analyzed using ImageJ, and the intensities at t=50 min are set as 1.0. Images were obtained at 50 min after addition of ascorbic acid (indicated as 00:50:00). Scale bar, 10 μm. (E) Live-cell imaging of cells transiently expressing cfSGFP2-col4a1 (green) and PDI-mCherry (red) at 8 h after addition of ascorbic acid. Confocal slices are piled on the Z-axis. White arrows indicate cfSGFP2-col4a1 secreted and deposited in the extracellular region. Scale bar, 20 μm. (F) Immunostaining of cfSGFP2-col4a1-expressing cells. After incubation in the presence of ascorbic acid for 3 days, cells were fixed and stained using anti-GFP (green) and anti-α1(IV) (magenta) antibodies without cell-permeabilization. White arrows are the same as in (E). Scale bars, 20 μm and 5 μm (inset).

To visualize ER-to-Golgi trafficking of procollagen IV, we constructed cfSGFP2-col4a1 by inserting cfSGFP2 at the N-terminus of the triple helical region of the proα1(IV) chain (Fig. 1A). We expressed cfSGFP2-col4a1 in the human sarcoma cell line HT-1080, which endogenously expresses collagen IV, because two proα1(IV) chains must assemble with a proα2(IV) chain to fold properly. Live-cell imaging revealed that cfSGFP2-col4a1 co-localized with the ER marker PDI-mCherry (Fig. 1B), confirming that cfSGFP2-col4a1 accumulated in the ER. Ascorbic acid was added to the culture medium to accelerate proline hydroxylation and thereby initiate ER-to-Golgi transport of procollagens (Berg and Prockop, 1973; Jimenez *et al.*, 1973; Rosenbloom *et al.*, 1973). Two hours after addition of ascorbic acid, cfSGFP2-col4a1 was detected in the Golgi area labeled with Golgi-BFP (Fig. 1C). Time-lapse imaging using fluorescence microscopy revealed that the GFP signal of procollagen IV gradually co-localized with the BFP signal of the Golgi complex at 80–95 min after addition of ascorbic acid and then rapidly disappeared from the Golgi region (Fig. 1D), indicating that cfSGFP2-col4a1 was transported from the ER to the Golgi complex over a similar period of time as endogenous procollagen IV, which was determined biochemically (Pihlajaniemi *et al.*, 1981). These results suggest that cfSGFP2-col4a1 synthesized in the ER was transported to the Golgi complex and then further sorted into the secretory pathway.

Fibrillar deposition of cfSGFP2-col4a1 was detected in the extracellular region at 8 h after addition of ascorbic acid (Fig. 1E). Immunostaining of cfSGFP2-col4a1 without cell permeabilization showed that the GFP signal accumulated outside of the cells similarly with the α1(IV) chain (Fig. 1F). These results suggest that the collagen IV trimer containing GFP-tagged proα1(IV) was deposited in the extracellular matrix in a similar manner as endogenous collagen IV. The transfected cfSGFP2-col4a1 cells expressed ~20–30% of the amount of endogenous procollagen IV (Suppl. Fig. 2A). Metabolic labeling confirmed that cfSGFP2-col4a1 was secreted into the medium as a trimer upon addition of ascorbic acid (Suppl. Fig. 2B) (Yoshikawa *et al.*, 2001). Taken together, these data indicate that cfSGFP2-col4a1 expressed in the ER was transported to the Golgi complex and then deposited in the extracellular matrix. Thus, cfSGFP2-col4a1 can be used to monitor the intracellular trafficking of endogenous procollagen IV.

### cfSGFP2-col4a1 is transported by vesicles with a diameter of ~300–500 nm

**Fig. 2:**
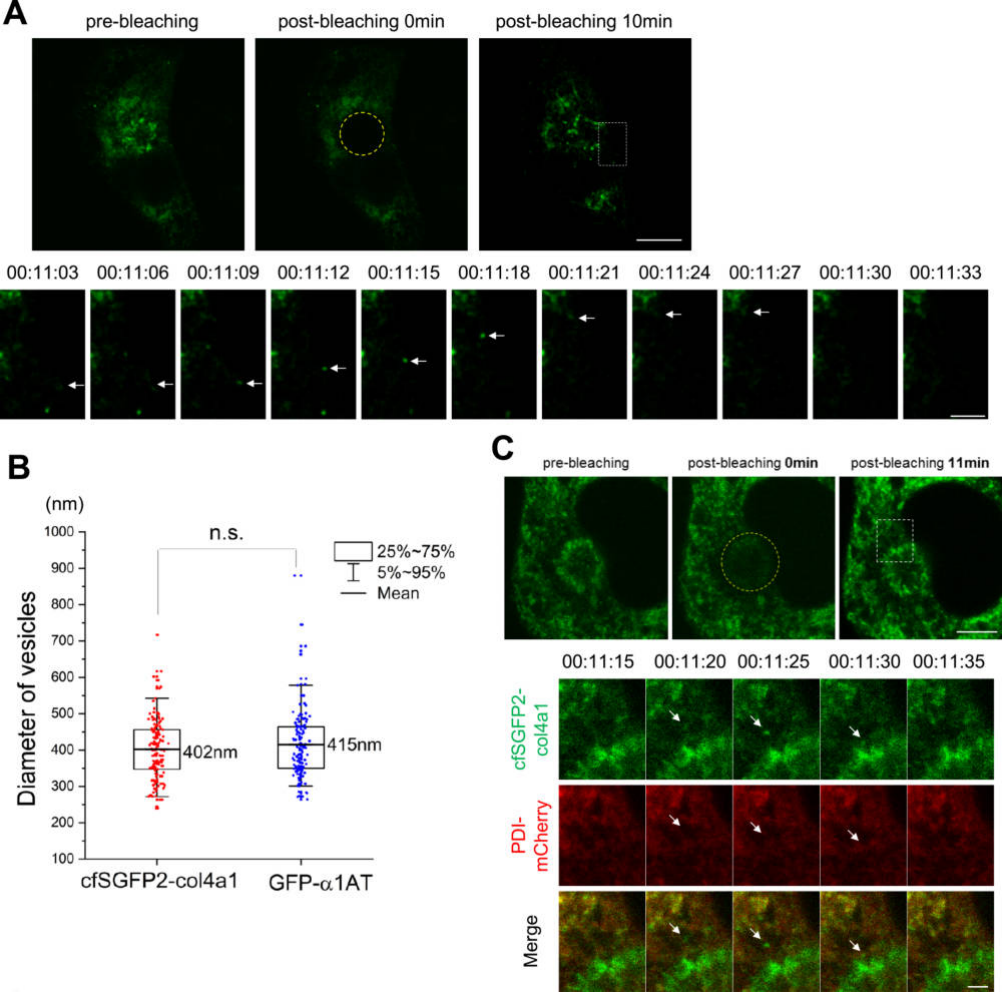
cfSGFP2-col4a1 is transported by vesicles from the ER to the Golgi. (A) Live-cell imaging of HT-1080 cells transiently expressing cfSGFP2-col4a1 (green) by confocal microscopy at 45 min after addition of ascorbic acid (pre-bleaching). After photo-bleaching the Golgi area (circled by yellow dotted line), time-lapse images were obtained every 3 sec (lower panels, white dotted boxed region) using a confocal microscope (Leica SP8) with the LIGHTNING package. Times denote the period after photo-bleaching. Arrows indicate a cfSGFP2-col4a1-containing vesicle trafficking from the ER to the Golgi. Scale bars, 10 μm (upper panels) and 5 μm (time-lapse). (B) Size distribution of GFP-positive vesicles moving toward the Golgi complex. The diameters of 153 cfSGFP2-col4a1-containing vesicles in five cells and 143 α1AT-containing vesicles in eight cells were measured in three independent experiments. ns: not significant (two-tailed Student’s t-test). (C) Same as in (A), except cells co-expressed cfSGFP2-col4a1 (green) and PDI-mCherry (red). Time-lapse images were acquired by fluorescence microscopy every 5 sec (lower panel) after photo-bleaching (t=00:00:00). Arrows indicate a cfSGFP2-col4a1-containing vesicle. Data are representative of five independent experiments. Scale bars, 7.5 μm and 2.5 μm (time-lapse).

To analyze how cfSGFP2-col4a1 is transported from the ER to the Golgi complex, we transfected HT-1080 cells with cfSGFP2-col4a1 and added ascorbic acid to the culture medium at 48 h after transfection. cfSGFP2-col4a1 appeared to be transported from the ER in vesicles; therefore, we photo-bleached the GFP signal in the Golgi region (Fig. 2A) to distinguish transport vesicles moving from the ER to the Golgi from those leaving the Golgi apparatus. After photo-bleaching, we recorded cfSGFP2-col4a1-containing vesicles merging with the Golgi complex over time (Fig. 2A and Movie 1). GFP-positive vesicles emerged from the ER and directly moved toward the Golgi area (Fig. 2A, lower panels). The GFP signal started to refill the Golgi apparatus at ~1 min 30 sec after photo-bleaching and was fully recovered at ~4 min, which was similar to the recovery period of GFP-α1AT after photo-bleaching (Suppl. Fig. 3 and Movie 2, see below).

We next measured the diameter of cfSGFP2-col4a1-containing vesicles and compared it with that of GFP-α1AT-containing carriers. α1AT is a conventional cargo transported by COPII vesicles (Venditti *et al.*, 2012) (Suppl. Fig. 3 and Movie 2). The diameter of both types of vesicles was ~300–500 nm, which was measured from the images obtained using a confocal microscope (Leica SP8) including the LIGHTNING package at ~140 nm resolution. The average diameter of vesicles containing cfSGFP2-col4a1 and GFP-α1AT was 402 and 415 nm, respectively, and this difference was not statistically significant (Fig. 2B). We did not observe megacarriers (Santos *et al.*, 2015) or large vesicles (Gorur *et al.*, 2017) exceeding ~600 nm in diameter. cfSGFP2-col4a1-containing vesicles did not co-localize with PDI-mCherry, confirming that they were not ER-associated vesicles (Fig. 2C and Movie 3). Collectively, these data suggest that cfSGFP2-col4a1 is transported from the ER to the Golgi complex by vesicles whose diameter is similar to that of conventional cargo carriers.

### Sar1 and SLY1/SCFD1 are required for export of procollagen IV from the ER

**Fig. 3:**
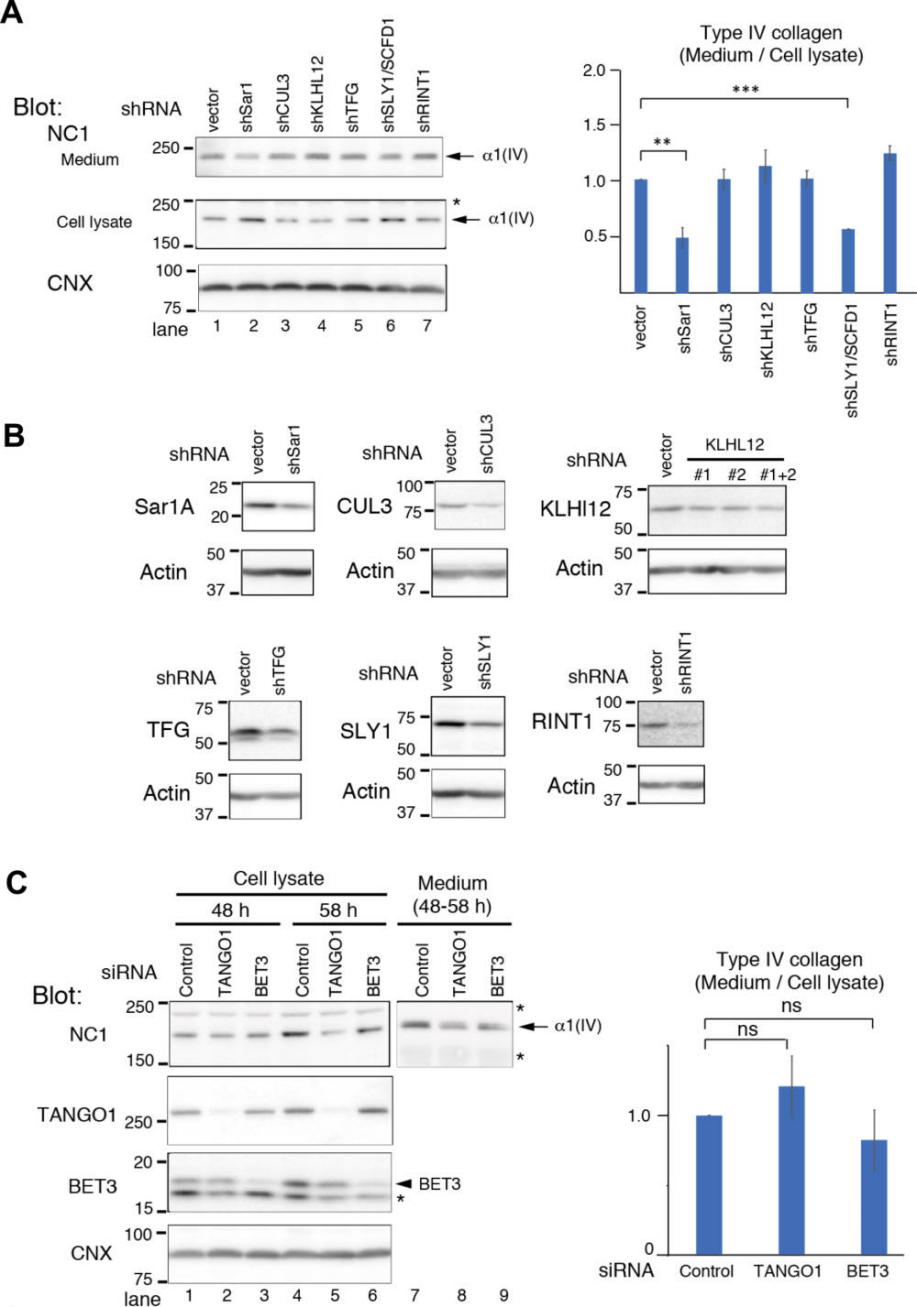
Knockdown analysis of factors required for procollagen IV export. (A) Twenty-four hours after the transfection with shRNAs, HT-1080 cells were cultured in the presence of ascorbic acid for additional 24 h. After changing the medium containing ascorbic acid and further incubating for 20 h, the cell lysate and culture medium were analyzed by immunoblotting with an anti-α1(IV) antibody. Secretion of collagen IV into the medium is quantified in the graph. The ratio of collagen between the culture medium and cell lysate was normalized to that in control cells. Calnexin (CNX) was used as a loading control. To knockdown KLHL12, the mixture of shKLHL12 #1 and #2 were used. Asterisk indicates a signal non-specifically detected by the anti-α1(IV) antibody. Error bars denote the SD of three independent experiments. **, P<0.01; ***, P<0.001 (two-tailed Student’s t-test, compared with control cells). (B) Western blot analysis of HT-1080 cells transfected with plasmids harboring shRNA targeting CUL3, KLHL12, TFG, SLY1/SCFD1, and RINT1 for 48 h. Actin was used as a loading control. (C) Same as in (A), except cells were transfected with siRNA for 48 h and were cultured in the presence of ascorbic acid for additional 10 h. Asterisks indicate signals non-specifically detected by the anti-α1(IV) and anti-BET3 antibodies. To calculate the ratio of collagens between the culture medium and cell lysate, the mean signal intensities of procollagen IV in the cell lysate at 48 h and at 58 h were used. Error bars denote the SD, n=3. ns, P>0.05 (two-tailed Student’s t-test).

Previous studies identified several factors required for ER-to-Golgi transport of procollagens VII and I (Cutrona *et al.*, 2013; Gorur *et al.*, 2017; Jin *et al.*, 2012; Johnson *et al.*, 2015; Mironov *et al.*, 2003; Nogueira *et al.*, 2014; Saito *et al.*, 2009; Stephens and Pepperkok, 2002; Venditti *et al.*, 2012). However, little is known about the factors necessary for such transport of collagen IV, which forms a distinct oligomeric structure. To investigate this, we knocked down proteins that reportedly affect transport of procollagens and analyzed secretion of endogenous collagen IV in HT-1080 cells by Western blotting. Knockout of TANGO1 and depletion of CUL3 inhibit secretion of collagen IV (Jin *et al.*, 2012; Wilson *et al.*, 2011). Transfection of small hairpin RNAs (shRNAs) targeting several factors reportedly required for collagen transport revealed that Sar1 and SLY1/SCFD1 effectively blocked secretion of endogenous procollagen IV (Fig. 3A). However, transfection of shRNAs targeting CUL3, KLHL12, TFG, and RINT1 did not inhibit secretion significantly (Fig. 3A), despite the fact that these shRNAs depleted their targets at least 50%, as revealed by Western blot analysis (Fig. 3B).

We also transfected cells with siRNA to knockdown TANGO1 and BET3, a component of the TRAPPII complex (Barrowman *et al.*, 2010), since the knockdown efficiencies of shTNAGO1 and shSedlin, another component of the TRAPPII complex, were insufficient. Transfection of siRNA efficiently knocked down TANGO1 and BET3 >90% and ~80–90%, respectively (Fig. 3C). We focused on the secretion of procollagen IV during the 48–58 h after siRNA treatment, because cytotoxicity started to appear in siTANGO1-transfected cells subjected to prolonged treatment. siTANGO1 treatment did not inhibit the secretion of procollagen IV (Fig. 3C), although procollagen IV secretion was slightly (~25%) but not significantly inhibited in siBET3-treated cells (Fig. 3C). To improve the knockdown efficiency of CUL3, KLHL12, and TFG, we transfected siRNA targeting these factors. Although the expression of CUL3 and KLHL12 were effectively reduced by >90% and ~70%, respectively, secretion of endogenous collagen IV was not affected (Suppl. Fig. 4). We could not analyze the effect of siTFG (McCaughey *et al.*, 2016) due to the apparent cytotoxic effect of the siRNA treatment.

Collectively, these results suggest that Sar1 and SLY1/SCFD1 are required for export of procollagen IV from the ER, while TANGO1, CUL3, KLHL12, TFG, RINT1, and BET3 are dispensable. To further confirm the requirement of Sar1 and SLY1/SCFD1 for the export of procollagen IV, we added back shRNA-resistant SAR1A and SAR1B, or SLY1/SCFD1 in cells treated with respective shRNAs. Transfection of the expression plasmids increased the amount of the target proteins, and partially rescued the secretion of procollagen IV (Suppl. Fig. 5), supporting the requirement of these factors.

Secretion of procollagen IV consists of two processes; ER-to-Golgi transport and Golgi-to-cell surface transport. To discriminate these two steps, we performed live-cell imaging and calculated the number of cells in which cfSGFP2-col4a1 was transported to the Golgi apparatus after the addition of ascorbate. In cells transfected with shSar1 and shSLY1/SCFD1, the frequency decreased to 33.7% and 52.9%, respectively, compared to the cells treated with control vector or shCUL3. Golgi-to-cell surface transport of cfSGFP2-col4a1 was also evaluated by time-lapse imaging using confocal microscopy. We were able to monitor the vesicles moving from the Golgi to the cell periphery for exocytosis, and the vesicle movements seemed comparable with the cells transfected with control vector, shSar1, or shSLY1/SCFD1 (Suppl. Fig. 6 and Movies 4–6). These results support that knockdown of Sar1 and SLY1/SCFD1 affected the ER-to-Golgi transport of procollagen IV.

### cfSGFP2-col4a1-containing vesicles do not co-localize with mCherry-ERGIC53

**Fig. 4:**
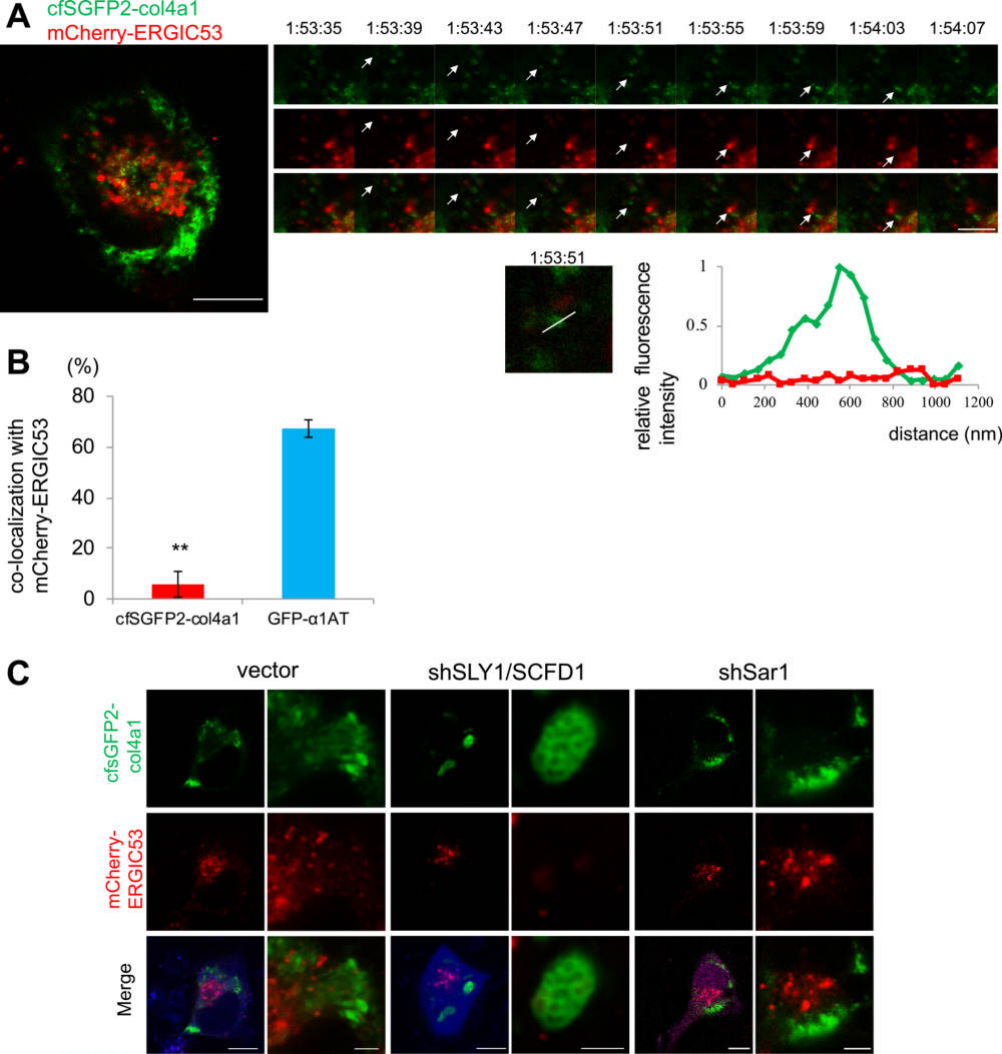
cfSGFP2-col4a1-containing vesicles do not colocalize with ERGIC53. (A) Live-cell imaging of HT-1080 cells transiently expressing cfSGFP2-col4a1 (green) and mCherry-ERGIC53 (red) after addition of ascorbic acid (t=00:00:00). Time-lapse images were acquired every 4 sec by confocal microscopy. Arrows indicate vesicles containing cfSGFP2-col4a1, but not mCherry-ERGIC53. The graph shows line-scan analysis of the fluorescence intensities of cfSGFP2-col4a1 (green) and mCherry-ERGIC53 (red) at t=01:53:51. Scale bars, 7.5 μm and 3.0 μm (inset). (B) HT-1080 cells were co-transfected with mCherry-ERGIC53 and cfSGFP2-col4a1 or GFP-α1AT. ER-to-Golgi transport vesicles were analyzed by confocal microscopy. Co-localization of mCherry-ERGIC53 with cfSGFP2-col4a1 and GFP-α1AT was analyzed on 176 vesicles from eight cells and 438 vesicles from 12 cells, respectively. Error bars denote the SEM of three independent experiments. *, P<0.05; **, P<0.01 (two-tailed Student’s t-test). (C) HT-1080 cells were transfected with a shRNA plasmid (blue and magenta), cfSGFP2-col4a1 (green), and mCherry-ERGIC53 (red), and incubated for 48 h. Live-cell imaging was performed by confocal microscopy at 18–20 h after addition of ascorbic acid to the culture medium. Data are representative of >50 cells in two independent experiments. In magnified views (right panels), only cfSGFP2-col4a1 and mCherry-ERGIC53 are shown in the merged figures. Scale bars, 10 μm (left panels) and 2.5 μm (right panels).

Formation of megacarriers for ER-to-Golgi transport of procollagen VII requires recruitment of ERGIC membranes (Santos *et al.*, 2015). To determine whether ERGIC membranes are incorporated into transport vesicles of collagen IV, we co-transfected cfSGFP2-col4a1 and mCherry-ERGIC53, and then performed time-lapse imaging. ERGIC53 is a cargo receptor that recognizes N-linked glycans and is an established marker of the ERGIC (Ben-Tekaya *et al.*, 2005; Itin *et al.*, 1996). Consistently, vesicles bearing GFP-α1AT, a cargo of ERGIC53 (Nyfeler *et al.*, 2008), often co-localized with mCherry-ERGIC53 (Suppl. Fig. 7 and Movie 7). By contrast, most cfSGFP2-col4a1-containing vesicles did not co-localize with mCherry-ERGIC53 (Fig. 4A and Movie 8). Quantification indicated that only 6.3% of cfSGFP2-col4a1-containing vesicles co-localized with mCherry-ERGIC53, in comparison with ~70% of GFP-α1AT-containing vesicles (Fig. 4B). Thus, we conclude that cfSGFP2-col4a1 transport vesicles do not incorporate membranes enriched with mCherry-ERGIC53.

Santos and colleagues reported that ERGIC-53-containing membranes are recruited to patches of procollagen VII in the ER, which are enlarged in ER SNARE-depleted cells (Santos *et al.*, 2015). We detected inhibition of ER-to-Golgi transport of procollagen IV and similar patches of cfSGFP2-col4a1 in the ER of shSar1 and shSLY1/SCFD1-expressing cells (Fig. 3A) and therefore examined whether ERGIC membranes were recruited to ER-retained cfSGFP2-col4a1 (Fig. 4C). We detected accumulation of procollagen IV in the ER of cells depleted of Sar1 or SLY1/SCFD1, which resembled to the patches reported for procollagen VII (Santos *et al.*, 2015). The appearance of the procollagen IV patches may look different to some extent (Fig. 4C), but we think that they are essentially the same in nature. However, mCherry-ERGIC53 was not recruited to patches of cfSGFP2-col4a1 in the ER or cfSGFP2-col4a1-containing vesicles even in shRNA-expressing cells. These results indicate that ERGIC membranes are not recruited during ER-to-Golgi transport of procollagen IV.

### cfSGFP2-col4a1-containing vesicles do not co-localize with mScarlet-RAB1A and -RAB1B

RAB1 is a small GTPase that regulates ER-to-Golgi transport (Nuoffer and Balch, 1994; Plutner *et al.*, 1991; Slavin *et al.*, 2011) and is an ERGIC marker (Saraste, 2016; Saraste *et al.*, 2009). Drosophila Tango1 interacts with RAB1 (Liu *et al.*, 2017), and knockdown of RAB1 inhibits export of collagen IV in Drosophila (Ke *et al.*, 2018). Hence, we analyzed co-localization of RAB1A and RAB1B with cfSGFP2-col4a1 in HT-1080 cells. Only 1.8% and 5.1% of cfSGFP2-col4a1-containing vesicles co-localized with mScarlet-RAB1A (Suppl. Fig. 8A and Movie 9) and mScarlet-RAB1B (Suppl. Fig. 8B and Movie 10), respectively. We detected cfSGFP2-col4a1-containing vesicles co-localized with or without mScarlet-RAB1B in the same cell (Movie 11). These results suggest that RAB1A and RAB1B are not involved in transport of procollagen IV in mammalian cells.

## Discussion

To visualize the intracellular trafficking and extracellular deposition of procollagen IV by live-cell imaging, we introduced cysteine-free SGFP2 at the N-terminus of the triple helical region of the α1(IV) chain (Fig. 1A). Collagen IV forms highly ordered lattice structures in the basement membrane. This is accomplished by reorganization of dimers and tetramers through association of the C-terminal NC1 and N-terminal 7S domains, respectively (Kühn, 1995; Kühn *et al.*, 1981; Timpl *et al.*, 1981). In addition, biosynthesis of procollagen trimers is initiated by assembly of C-terminal propeptides (Davis *et al.*, 1989; Dölz *et al.*, 1988). Thus, to avoid steric hindrance during trimer assembly in the ER and meshwork formation after secretion, we inserted the fluorescent protein at the N-terminus of the triple helical region. Furthermore, cysteine-free SGFP2 was used to avoid thiol-mediated interactions with non-native cysteine residues of collagen IV and other molecules during oxidative folding (Suzuki *et al.*, 2012). By expressing cfSGFP2-col4a1 in HT-1080 cells, which endogenously express collagen IV, we successfully detected the synthesis, secretion, and extracellular deposition of the GFP-tagged α1(IV) chain, which enabled us to analyze ER-to-Golgi transport of procollagen IV in living cells. The ER-to-Golgi transport by vesicles were mostly observed at ~1–2 h after the addition of ascorbate. Additional transport carriers were also detected after the periods, probably due to the large pool of cfSGFP2-col4a1 accumulated in the ER (Fig. 1D). The transfection efficiency of cfSGFP2-col4a1 in HT-1080 cells was ~70%. Considering that the amount of cfSGFP2-col4a1 was ~20–30% of the endogenous procollagen IV (Suppl. Fig. 2A), each cell appeared to express cfSGFP2-col4a1 less than that of the endogenous procollagen IV on average.

We detected movement of cfSGFP2-col4a1-containing vesicles from the ER to the Golgi complex by confocal microscopy after photo-bleaching the Golgi area (Fig. 2A). The diameter of these vesicles was ~300–500 nm (average 402 nm), which was similar to that of conventional GFP-α1AT-containing carriers (average 415 nm). COPII-coated conventional cargoes fuse to form larger vesicles of ~300–500 nm shortly after budding from the ERES (Stephens *et al.*, 2000), which is consistent with the GFP-α1AT-containing carriers we detected in this study. Compared to the length (~400 nm) of procollagen IV, the diameter of cfSGFP2-col4a1-containing vesicles appears smaller. However, a recent study demonstrated that collagens I, II, and III are more flexible than previously described and behave as an “equilibrated curved worm-like chain” (Rezaei *et al.*, 2018). In addition, the central zone of collagen IV contains multiple sequences that interrupt the triple helix such as Gly-Pro-Gly, and this protein exhibits various degrees of flexibility along the chain (Bella *et al.*, 2006; Hofmann *et al.*, 1984). Hence, it is not mechanically unfavorable for ~400 nm-long procollagen IV (Bachinger *et al.*, 1982) to fit into the vesicles that we detected. This is reminiscent of enlarged COPII vesicles with a diameter of ~300–500 nm carrying procollagen I (Gorur *et al.*, 2017; Jin *et al.*, 2012), although much larger vesicles have also been described. The diameter and characteristics of transport vesicles containing GFP-labeled-proα1(I) collagen (Stephens and Pepperkok, 2002) are similar to those of the transport vesicles reported in the current study. However, the vesicles containing C-terminally GFP-tagged proα1(I) (Stephens and Pepperkok, 2002) were recently reported to move poorly (McCaughey *et al.*, 2018).

Importantly, cfSGFP2-col4a1-containing vesicles did not fuse with those containing mCherry-ERGIC53 (Fig. 4A) or with mScarlet-RAB1A or -RAB1B (Suppl. Fig. 8), suggesting that the properties of these transport vesicles are distinct from those of conventional GFP-α1AT-containing carriers (Suppl. Fig. 3), which recruit ERGIC membranes. Furthermore, this also suggests that procollagen IV does not require megacarriers, which are used to export collagen VII and are generated by fusion with ERGIC membranes (Santos *et al.*, 2015). The ERGIC is a dynamic or stable sorting hub for anterograde or retrograde transport at the ER-Golgi interface; therefore, the lack of an ERGIC component in cfSGFP2-col4a1-containing vesicles suggests that these carriers are devoid of molecules required for membrane tethering/fusion with the intermediate compartment. We therefore examined the involvement of RAB1, which regulates this process. We hypothesized that the large size of tubular structures containing RAB1 (Marie *et al.*, 2009; Sannerud *et al.*, 2006) may be favorable for accommodating procollagens; however, these structures did not contain cfSGFP2-col4a1. Although RAB1 is abundant in COPII-coated ERES, these results suggest that ER export of cfSGFP2-col4a1 uses unique vesicles that are directly transported to the cis-Golgi network. It is tempting to speculate that this may be related to the maturation mechanisms of procollagen IV. Recycling in the early secretory pathway via the ERGIC assists the proper maturation of small cargo such as α1AT because the ERGIC also contains molecules for quality control such as UDP-glucose:glycoprotein glucosyltransferase (Zuber *et al.*, 2001). However, recycling in the early secretory pathway facilitated by ERGIC membranes may be too inefficient for a bulky and rigid protein such as collagen IV.

The factors required for export of procollagen IV from the ER were identified by knockdown experiments (Fig. 3, Suppl. Figs. 4 and 5). Western blot analysis of endogenous collagen IV in HT-1080 cells indicated that Sar1 and SLY1/SCFD1 are necessary for transport of procollagen IV, but not TANGO1, CUL3, KLHL12, TFG, and RINT1. These results suggest that procollagen IV is transported out of the ER via a different mechanism from that of procollagen VII and I. We were surprised that TANGO1 was not required for the transport of procollagen IV, because TANGO1 is a multifunctional protein that resides at ERES (Saito *et al.*, 2009), and the secretion of collagen IV and other types of collagen is inhibited in TANGO1-knockout mice (Wilson *et al.*, 2011). TANGO1 is also required for the transport of procollagen IV in Drosophila (Pastor-Pareja and Xu, 2011), and interaction of procollagen IV with TANGO1 through HSP47 has also been reported (Ishikawa *et al.*, 2016). However, precise requirement of TANGO1 in human procollagen IV transport is not clear at present. Because intracellular procollagen IV decreased 58 h after siTANGO1 transfection (Fig. 3C), we suspected appearance of non-specific cytotoxicity by a prolonged treatment of siTANGO1. Further analysis will be required to elucidate the function of TANGO1 in the synthesis and transport of procollagens. In addition to TANGO1, TFG, SLY1/SCFD1, STX18, and NRZ (NBAS, RINT1, ZW10) are known to be involved in fusion of the ER to the ERGIC (McCaughey *et al.*, 2016; Nogueira *et al.*, 2014; Raote *et al.*, 2018; Santos *et al.*, 2015). Among these factors, we found that only SLY1/SCFD1 was required for ER export of procollagen IV, although we could not analyze the requirement for STX18 due to its low knockdown efficiency. This finding points to existence of a unique facility for the export of procollagen IV. Procollagen IV likely exits the ER in enlarged COPII vesicles, but without engaging the function of the CUL3-KLHL12 ubiquitin-ligase complex, which was required to enlarge the COPII vesicles to accommodate procollagen I (Gorur *et al.*, 2017; Jin *et al.*, 2012). Recently, it was reported that CUL3-KLHL12 is involved in the recognition of misfolded procollagens at ERES (Omari *et al.*, 2018). In this scenario, CUL3-KLHL12 is not required for the ER-to-Golgi transport of procollagens.

Collectively, based on live-cell imaging, we propose a novel pathway in which procollagen IV is transported from the ER to the Golgi complex in large COPII vesicles with a diameter of ~300–500 nm (average ~400 nm). These vesicles may fuse with each other just after budding from the ER, but do not fuse with ERGIC membranes. Furthermore, cfSGFP2-col4a1 will be useful to analyze collagen secretion from the Golgi complex and to study the dynamics of collagen IV in the extracellular matrix.

## Abbreviations

α1AT: α_1_-antitrypsin
cfSGFP2: cysteine-free SGFP2
COPII: coat protein complex II
ER: endoplasmic reticulum
ERES: ER exit site
ERGIC: ER-Golgi intermediate compartment
HSP47: heat shock protein 47
shRNA: small hairpin RNA
siRNA: small interfering RNA
