# Supplementary material for: Visualization of Procollagen IV Reveals ER-to-Golgi Transport by ERGIC-independent Carriers
Source: Cell Struct Funct. 2020 Jun 18;45(2):107–19. doi: 10.1247/csf.20025 (PMC10511052; doi:10.1247/csf.20025)
Supplement: Supplementary file 1 — Supplemental Figure 1 [file csf_45_20025_1.pdf]

Supplemental Figure 1

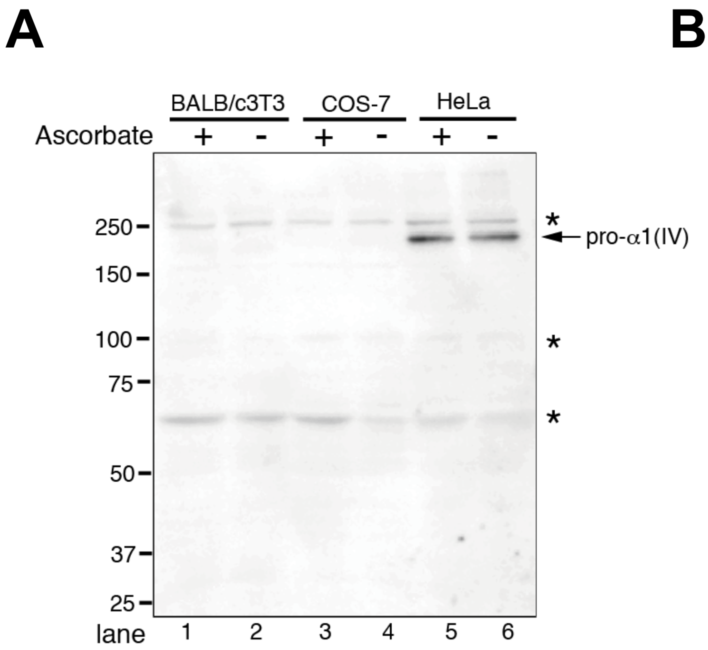

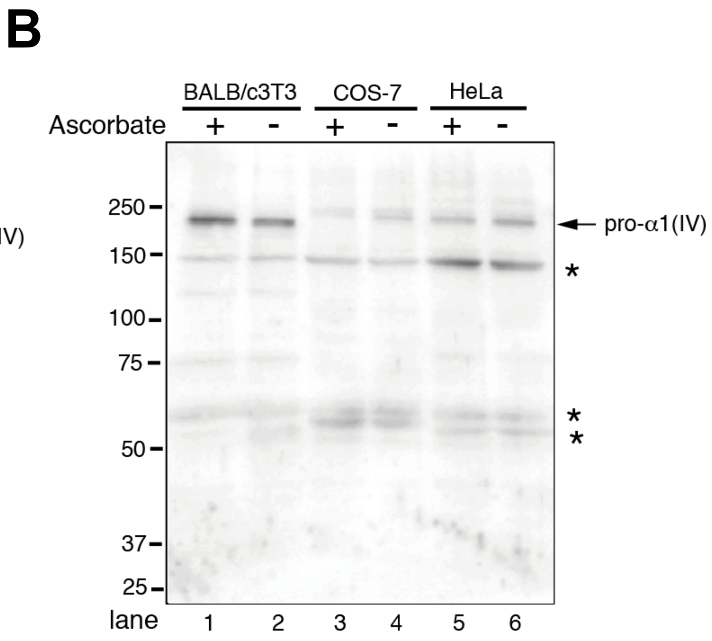

Supplemental Figure 1. Validation of anti-NC1 antibody.
Western blot analysis of BALB/c3T3 (mouse), COS-7 (African green monkey), and Hela (human) cell lysates using an antibody against the NC1 domain of the human procollagen IV α1 chain (A), compared with that detected by an antibody raised against mouse collagen IV (purified from EHS tumor) (B). Antibodies were kindly provided by Dr. Sasaki (Oita University, Japan). Cell lysates were separated by 8% SDS-PAGE under reducing conditions.
