# Supplementary material for: Visualization of Procollagen IV Reveals ER-to-Golgi Transport by ERGIC-independent Carriers
Source: Cell Struct Funct. 2020 Jun 18;45(2):107–19. doi: 10.1247/csf.20025 (PMC10511052; doi:10.1247/csf.20025)
Supplement: Supplementary file 2 — Supplemental Figure 2 [file csf_45_20025_2.pdf]

Supplemental Figure 2

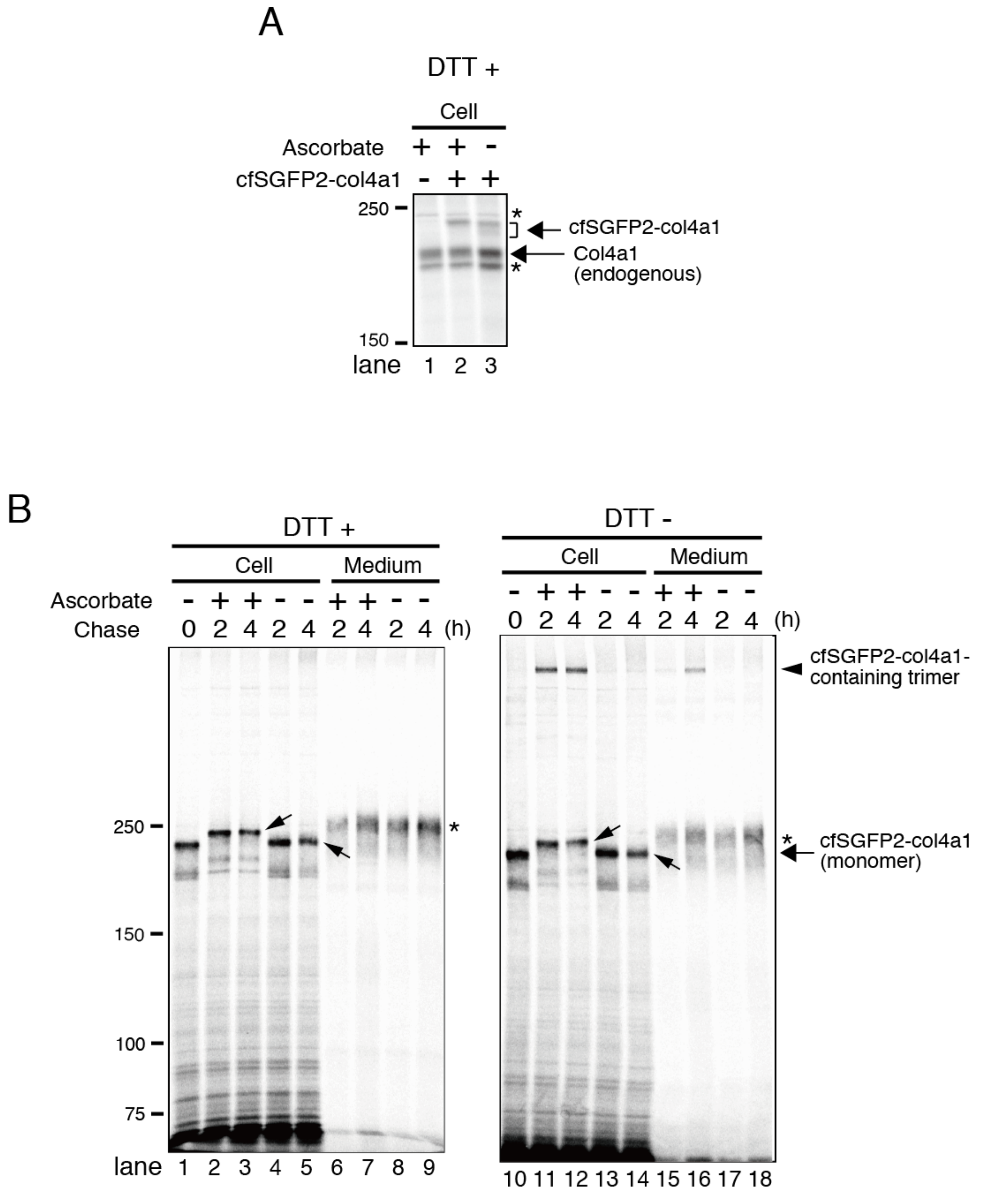

Supplemental Figure 2. cfSGFP2-col4a1 is secreted into the culture medium as a trimer.
(**A**)Forty-eight hours after transfection with cfSGFP2-col4a1, HT-1080 cells were metabolically labeled with ^{35}S-methionine/cysteine for 24 h. The cell lysates were immunoprecipitated using an anti-α1(IV) antibody and proteins were separated by 5% SDS-PAGE under reducing condition. Specific signals were detected by exposing the gel to an imaging plate. A representative result of three independent experiments is shown. The long and short arrows indicate endogenous procollagen IV and cfSGFP2-col4a1, respectively. The asterisks indicate non-specific signals detected by the anti-α1(IV) antibody.
(**B**) Thirty-eight hours after transfection with cfSGFP2-col4a1, HT-1080 cells were metabolically labeled with ^{35}S-methionine/cysteine for 90 min and chased for the indicated periods in the presence or absence of ascorbate. Cell lysate and medium were immunoprecipitated using an anti-GFP antibody and separated by 5% SDS-PAGE under reducing (DTT+) and non-reducing (DTT-) conditions. Arrows indicate the cfSGFP2-col4a1 monomer. The arrowhead indicates the procollagen IV trimer containing cfSGFP2-col4a1. Asterisks indicate non-specific signals detected by the anti-GFP antibody.
