# Supplementary material for: Visualization of Procollagen IV Reveals ER-to-Golgi Transport by ERGIC-independent Carriers
Source: Cell Struct Funct. 2020 Jun 18;45(2):107–19. doi: 10.1247/csf.20025 (PMC10511052; doi:10.1247/csf.20025)
Supplement: Supplementary file 3 — Supplemental Figure 3 [file csf_45_20025_3.pdf]

# Supplemental Figure 3

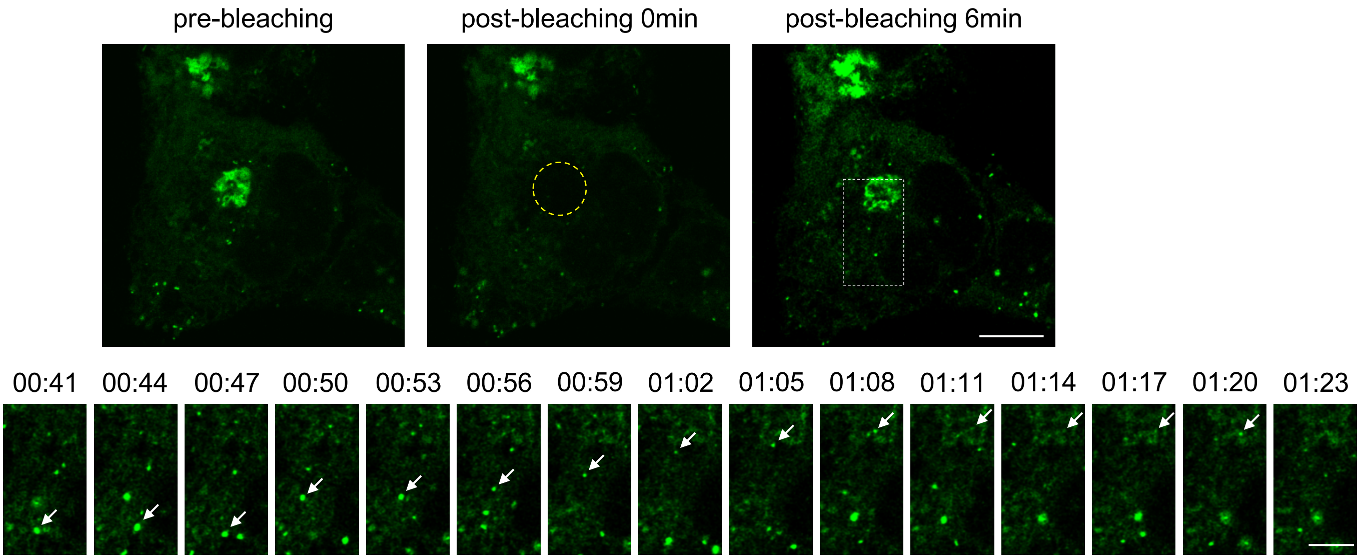


**Supplemental Figure 3. α1AT-GFP is transported by vesicles from the ER to the Golgi.**
Live-cell imaging of HT-1080 cells transiently expressing α1AT-GFP (green) by confocal microscopy (pre-bleaching). After photo-bleaching the Golgi area (circled by yellow dotted line, t=00:00), time-lapse images were acquired every 3 sec (lower panels) using a confocal microscope (Leica SP8) with the LIGHTNING package. Arrows indicate an α1AT-GFP-containing vesicle trafficking from the ER to the Golgi. A representative result of three independent experiments with 143 vesicles from 8 cells (shown in Fig. 2B). Scale bars, 10 μm (upper panels) and 5 μm (time-lapse).
