# Supplementary material for: Visualization of Procollagen IV Reveals ER-to-Golgi Transport by ERGIC-independent Carriers
Source: Cell Struct Funct. 2020 Jun 18;45(2):107–19. doi: 10.1247/csf.20025 (PMC10511052; doi:10.1247/csf.20025)
Supplement: Supplementary file 4 — Supplemental Figure 4 [file csf_45_20025_4.pdf]

Supplemental Figure 4

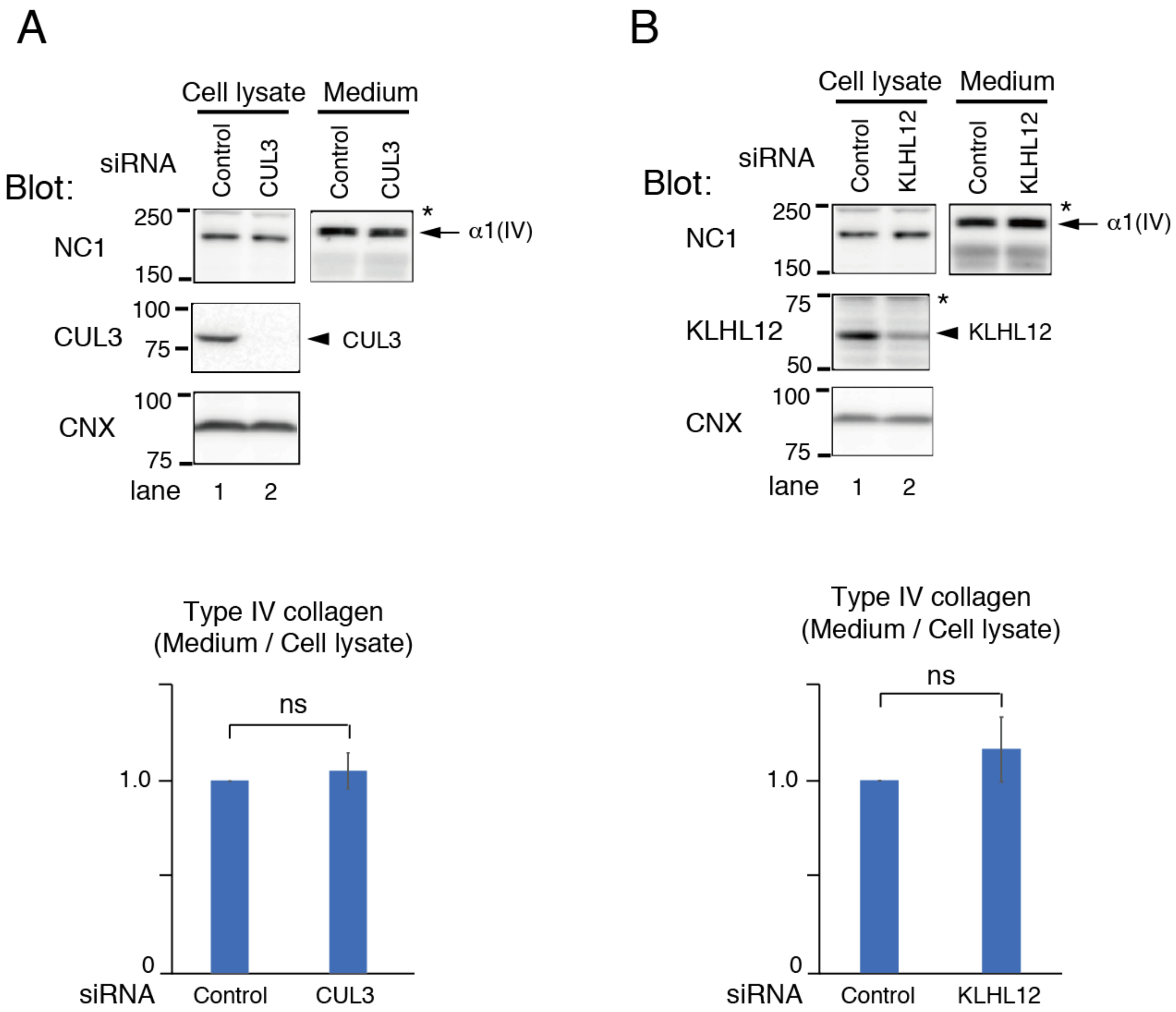

Supplemental Figure 4. siRNA-mediated knockdown analysis of CUL3 and KLHL12 for procollagen IV export.
(**A**) Forty-eight hours after the transfection with siRNA targeting CUL3, HT-1080 cells were cultured in the presence of ascorbic acid for additional 20 h. The cell lysate and culture medium were analyzed by immunoblotting with indicated antibodies. Secretion of collagen IV into the medium was quantified. The ratio of collagen between the culture medium and cell lysate was normalized to that in control cells. Calnexin (CNX) was used as a loading control. Asterisks indicate signals non-specifically detected by the anti-α1(IV) antibody. Error bars denote the SD of 3 independent experiments. ns, $P > 0.05$ (two-tailed Student's t-test).
(**B**) Same as in (A), except siRNA targeting KLHL12 was used and ascorbic acid was added 24 h after the transfection to avoid cytotoxic effect caused by the prolonged treatment of siRNA. Asterisks indicate bands non-specifically detected by the anti-α1(IV) and anti-KLHL12 antibodies.
