# Supplementary material for: Visualization of Procollagen IV Reveals ER-to-Golgi Transport by ERGIC-independent Carriers
Source: Cell Struct Funct. 2020 Jun 18;45(2):107–19. doi: 10.1247/csf.20025 (PMC10511052; doi:10.1247/csf.20025)
Supplement: Supplementary file 5 — Supplemental Figure 5 [file csf_45_20025_5.pdf]

Supplemental Figure 5

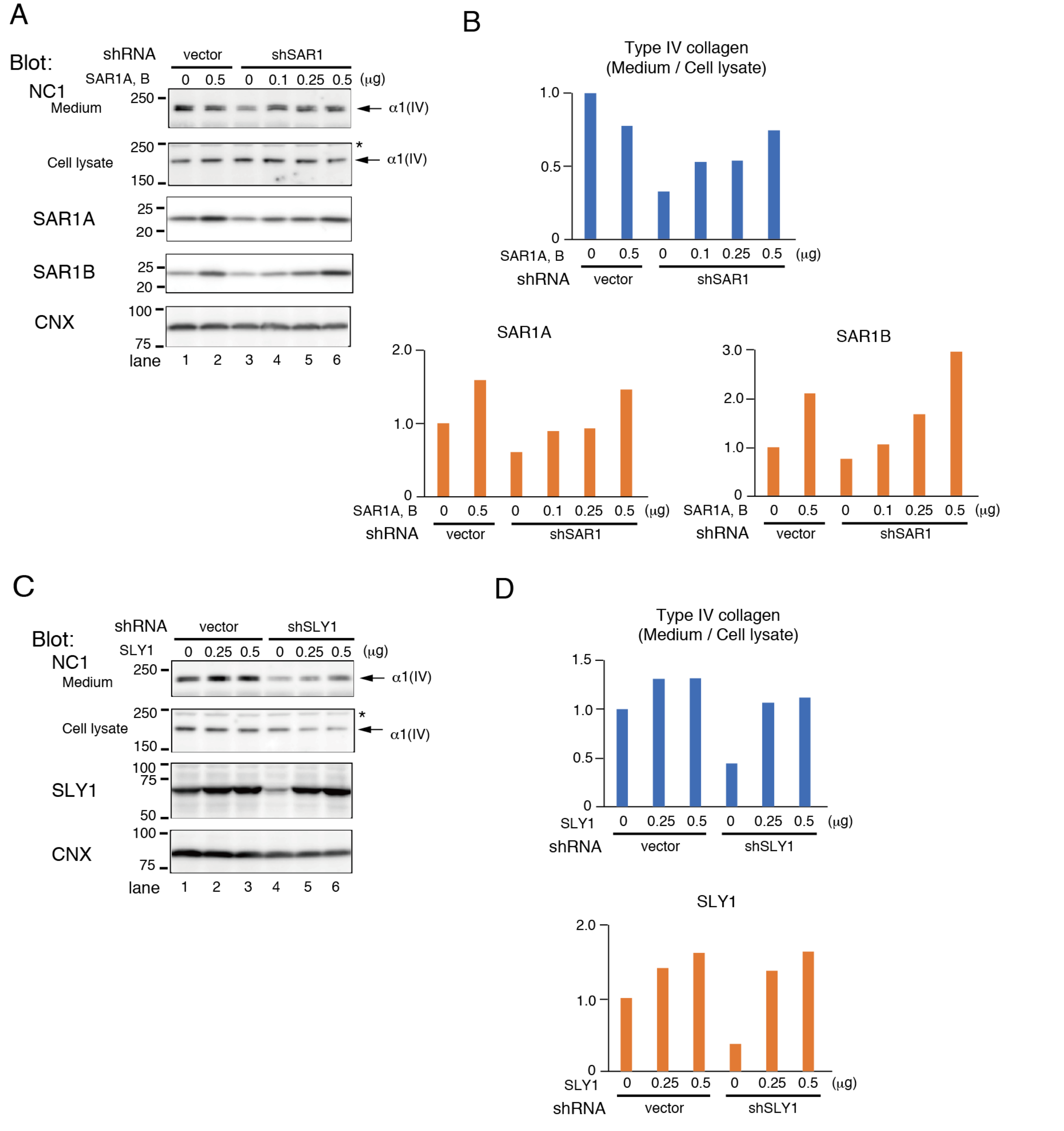

Supplemental Figure 5. Expression of shRNA-resistant SAR1A and SAR1B, or SLY1/SCFD1 restores secretion of procollagen IV in shRNA-treated cells.

(**A**) Cells were transfected with both shSar1 (1 μg) and shRNA-resistant SAR1A and SAR1B at the indicated amounts. Forty-eight hours after transfection, cells were cultured in the presence of ascorbic acid for additional 20 h. The cell lysate and culture medium were analyzed by immunoblotting with indicated antibodies. Asterisks indicate signals non-specifically detected by the anti-α1(IV) antibody. Calnexin (CNX) was used as a loading control. A representative result of four independent experiments is shown.

(**B**) Secretion of collagen IV into the medium and the expression levels of SAR1A and SAR1B in (A) are quantified. The ratio of collagen between the culture medium and cell lysate was normalized to that in control cells.

(**C**) Same as in (A), except shSLY1/SCFD1 and shRNA-resistant SLY1/SCFD1 were used.

(**D**) Secretion of collagen IV into the medium and the expression level of SLY1/SCFD1 in (C) are quantified. The ratio of collagen between the culture medium and cell lysate was normalized to that in control cells.
