# Supplementary material for: Visualization of Procollagen IV Reveals ER-to-Golgi Transport by ERGIC-independent Carriers
Source: Cell Struct Funct. 2020 Jun 18;45(2):107–19. doi: 10.1247/csf.20025 (PMC10511052; doi:10.1247/csf.20025)
Supplement: Supplementary file 6 — Supplemental Figure 6 [file csf_45_20025_6.pdf]

Supplemental Figure 6

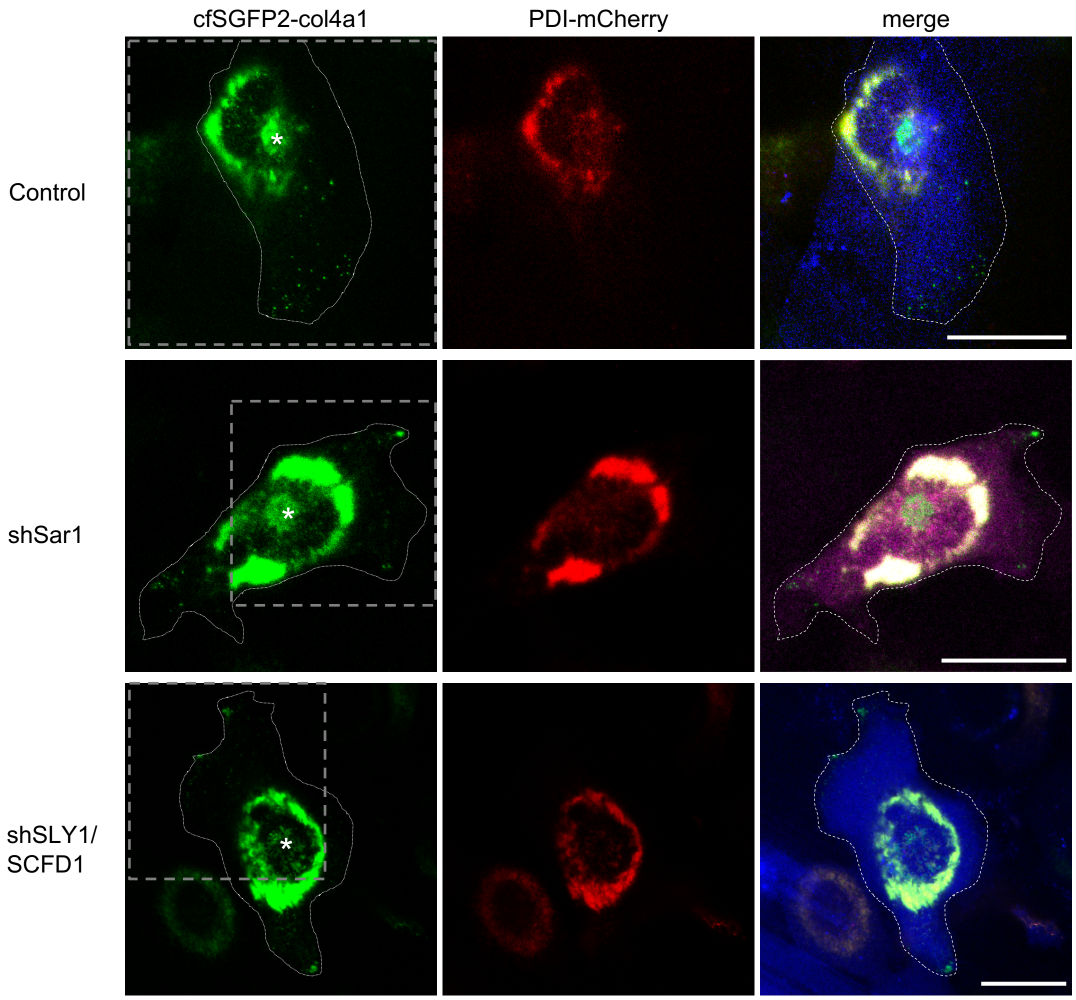

Supplemental Figure 6. Golgi-to-plasma membrane transport of cfSGFP2-col4a1.
Live-cell imaging of HT-1080 cells transfected with a shRNA plasmid (blue and magenta), cfSGFP2-col4a1 (green), and PDI-mCherry (red) were incubated for 48 h. After addition of ascorbic acid, time-lapse images in Movie 4-6 (gray dotted boxed region) were acquired every 4 sec by confocal microscopy setting the pinhole to 7.0 Airy Unit. Asterisks indicate the Golgi apparatus. A representative result of two independent experiments with 6, 3, and 4 cells, for control, shSar1-, and shSLY1/SCFD1-treated cells, respectively. Scale bars, 30 µm.
