# Supplementary material for: Visualization of Procollagen IV Reveals ER-to-Golgi Transport by ERGIC-independent Carriers
Source: Cell Struct Funct. 2020 Jun 18;45(2):107–19. doi: 10.1247/csf.20025 (PMC10511052; doi:10.1247/csf.20025)
Supplement: Supplementary file 7 — Supplemental Figure 7 [file csf_45_20025_7.pdf]

# Supplemental Figure 7

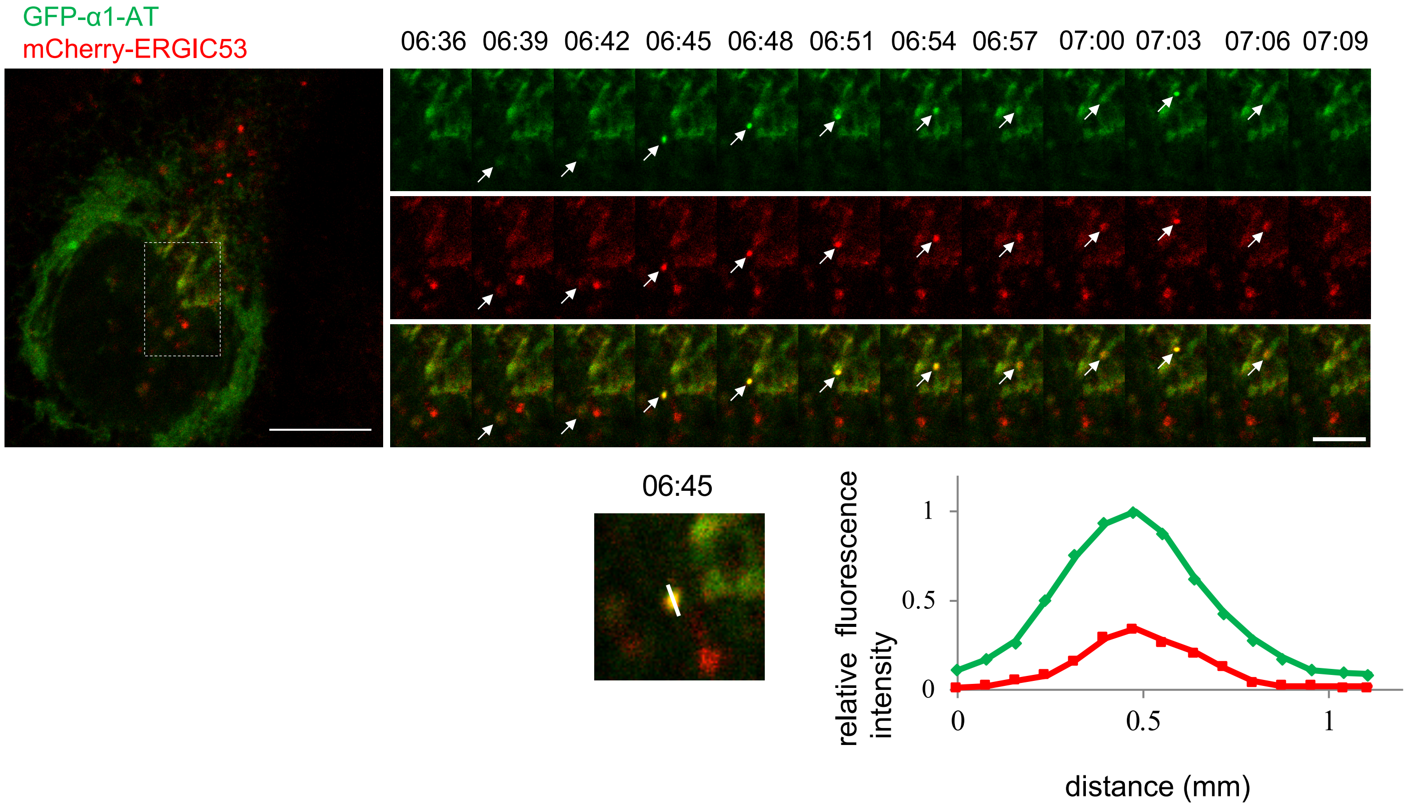


**Supplemental Figure 7. GFP-α1AT-containing vesicles co-localize with ERGIC53.**
Live-cell imaging of HT-1080 cells transiently expressing GFP-α1AT (green) and mCherry-ERGIC53 (red) at 24 h after transfection. After photo-bleaching the Golgi region (t=00:00), time-lapse images were acquired every 3 sec by confocal microscopy. Arrows indicate vesicles containing GFP-α1AT and mCherry-ERGIC53. The graph shows line-scan analysis of the fluorescence intensities of GFP-α1AT and mCherry-ERGIC53 at t=06:45. A representative result of three independent experiments with 438 vesicles from 12 cells. Scale bars, 10 μm and 5 μm (time-lapse).
