# Supplementary material for: Visualization of Procollagen IV Reveals ER-to-Golgi Transport by ERGIC-independent Carriers
Source: Cell Struct Funct. 2020 Jun 18;45(2):107–19. doi: 10.1247/csf.20025 (PMC10511052; doi:10.1247/csf.20025)
Supplement: Supplementary file 8 — Supplemental Figure 8 [file csf_45_20025_8.pdf]

Supplemental Figure 8

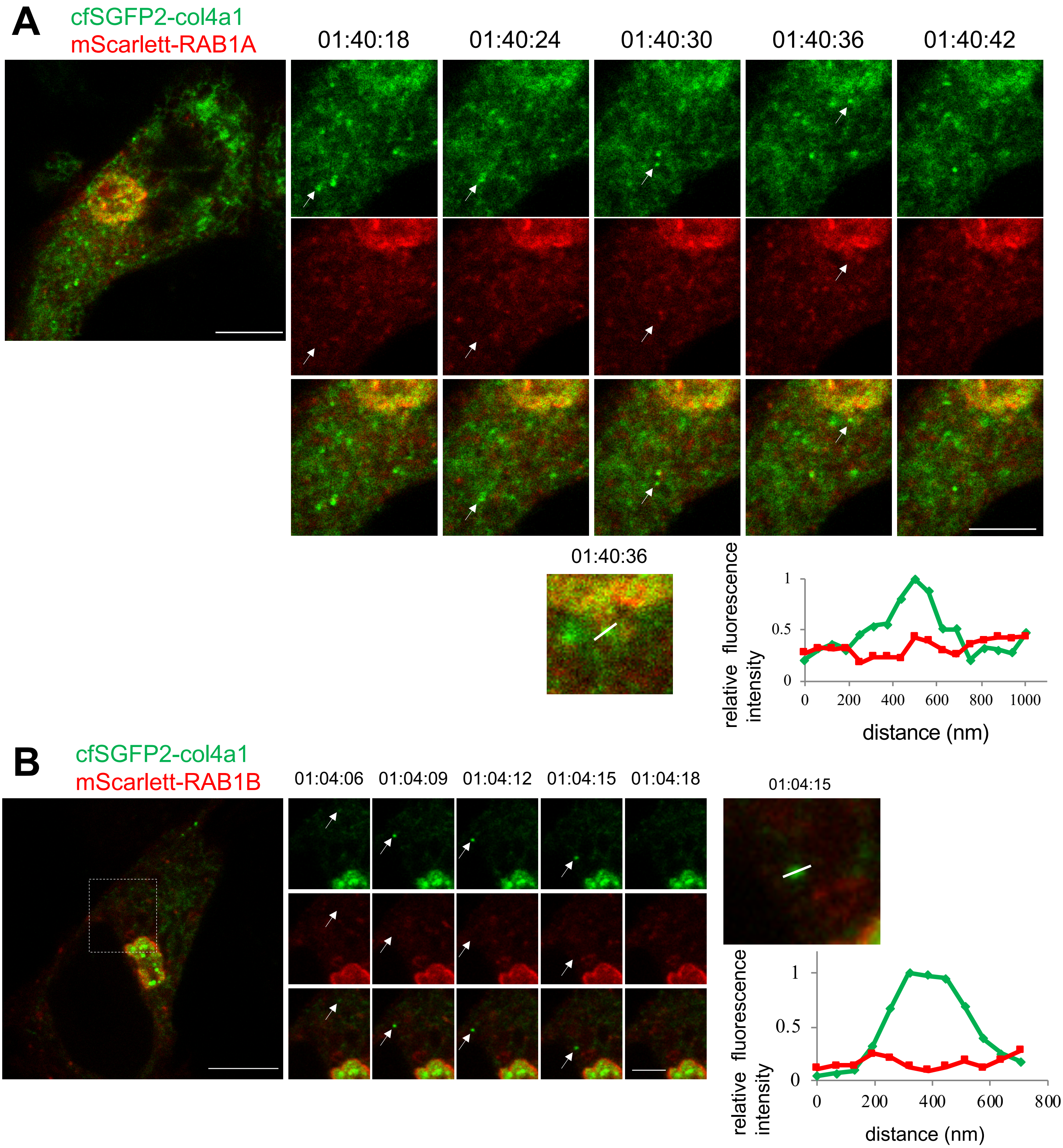

Supplemental Figure 8. cfSGFP2-col4a1-containing vesicles do not colocalize with RAB1A or RAB1B.
(**A**) Live-cell imaging of HT-1080 cells transiently expressing cfSGFP2-col4a1 (green) and mScarlet-RAB1A (red) at 48 h after transfection. Time-lapse images were acquired every 6 sec by confocal microscopy starting at 1 h after addition of ascorbic acid (t=01:00:00). Arrows indicate vesicles containing cfSGFP2-col4a1. Among 171 cfSGFP2-col4a1-containing vesicles from eight cells in two independent experiments, only three vesicles (1.8%) co-localized with mScarlet-RAB1A. Line-scan analysis shows the fluorescence intensities of cfSGFP2-col4a1 and mScarlet-RAB1A at t=01:40:36. Scale bars, 7.5 μm and 3 μm (time-lapse).
(**B**) Same as in (A), except mScarlet-RAB1B (red) was transfected. Time-lapse images were acquired every 3 sec starting at 1 h after addition of ascorbic acid (t=01:00:00). Among 169 cfSGFP2-col4a1-containing vesicles from ten cells in three independent experiments, only nine vesicles (5.1%) co-localized with mScarlet-RAB1B. Line-scan analysis shows the fluorescence intensities of cfSGFP2-col4a1 and mScarlet-RAB1B at t=01:04:15. Arrows and scale bars are the same as in (A).
